# Bibliographical Notices

**Published:** 1853-03

**Authors:** 


### BIBLIOGRAPHICAL NOTICES.

*Report of the Pennsylvania Hospital for the Insane, for the year* 1852. By THOMAS S. KIRKBRIDE, M. D., Physician to the Hospital.

Dr. Kirkbride's Twelfth Annual Report presents a most gratifying account of the noble Institution under his charge. "It closes its twelfth year," we learn, "in a state of high prosperity; its buildings about as extensive as are desirable, nearly every room in all its wards constantly required to accommodate those who resort to it for relief, and its means of adding to the comfort and happiness of its patients, and carrying out a liberal course of treatment, steadily rising in character, and increasing in number and efficiency."

The most important improvement made in the Hospital, during the past year, is the introduction of *gas* for lighting the Hospital buildings. It is brought a distance of nearly two miles from the Philadelphia Gas Works ; and its "convenience, cleanliness, safety, and economy" have been made so apparent, that Dr. Kirkbride views it as really indispensable in an Institution of the kind. And he considers it, besides, an important remedy :—

"The effect of cheerful, well-lighted halls and parlors is most striking upon every class of patients, as will readily be acknowledged by all who have watched their appearance or heard their remarks, where an improved mode of lighting has been introduced. There can be scarce anything more dreary about such establishments at night, than the appearance of their long halls so dimly lighted as barely to prevent persons running against each other, or parlors in which no one can read but in immediate proximity to the feeble lamp. When it is remembered, too, that the period during which this state of things exists, is often for at least six hours out of the sixteen that patients are out of bed, it may readily be understood how important it is to change all this gloom into cheerfulness, and to substitute a bountiful supply of gas-light for the obscurity commonly connected with ordinary lamps."

Institutions, distant from large cities, "can have the gas made on the premises, about as cheap as it can be ordinarily bought, and without difficulty, risk, or trouble of any kind."

Among the points to which Dr. Kirkbride particularly adverts in the Report before us, is the importance of a farm and garden as an appendage to an Hospital for the Insane.

"No new Institution, I feel quite confident," he says, "will hereafter be put up on less than one hundred acres, which amount seems to be indispensable to give the proper degree of privacy, to provide sufficient means of labor and of exercise, and to furnish the supplies that are required. It also seems well established that a wise foresight, sound views of the objects and real capabilities of Hospitals for the Insane, and true economy, should lead the older Institutions, less fortunately situated, at the earliest possible day, to dispose of their present establishments, and to provide new ones in more favorable locations. The experiment of transferring a Hospital for the Insane from a city to the country, of substituting new for old buildings, and having more than a hundred instead of a few acres of land, was made by the Pennsylvania Hospital in 1841, and I presume no one will now be found to doubt the propriety of the course adopted, nor to raise the question, whether the results of that change, costly as it was, have not been such as to justify all the expense that was incurred."

Among the desirable improvements that have been lately added to the Institution, we notice with pleasure a *calisthenium*, intended to give the female patients the free use of calisthenic exercises. To free muscular exercise in the open air, Dr. Kirkbride attaches prominent importance as a therapeutic resource in the treatment of nervous disorders—quite as essential for the female as the male sex. "Muscles versus Nerves" is the motto which has been pleasantly adopted for the new calisthenium; and Dr. Kirkbride very aptly introduces the following strain of remarks "from an intelligent patient, in noticing this motto:"

"She very naturally inquired whether, if it was true that the use and development of the muscles kept down nervous irritability and removed nervous diseases, some very marked change had not come over the views of professional men and hospital practice, for that, in her early experience among the excited class, the grand object seemed to be to keep down and weaken the muscles, and let the nerves take care of themselves. Or else what could mean the varied and systematic modes of direct depletion, with the low diet, and, above all, the studied efforts to keep quiet these same muscles, either in a forced recumbent position, or worse, if upright, in that machine, whose title seems to have been framed in bitter irony of its real effects, the tranquillizing chair.' It seemed as though some grave surgical disease might be supposed to exist in every part of the body, which the slightest movements of the muscles would aggravate. The muscles of the tongue, however, were left at liberty, and full advantage was generally taken of this partial freedom, to give them, at least, abundant exercise, to the great annoyance of all in the vicinity. This criticism on that mode of treatment could hardly have been more pointed or more just. Much as it might reduce the muscular system, the experience of this, and of most other patients, would certainly be, that the reduction brought with it neither tranquillity to the house, comfort to the afflicted, nor composure to the disturbed mind."

We close this acceptable report, with the warmest feelings of gratification at the renewed evidence which it offers, of unceasing devotion to the physical comfort and therapeutic management of the insane.

*On the Employment of Iodide of Potassium as a Remedy for the Affections caused by Lead and Mercury.* By M. MELSENS. Translated by WILLIAM BUDD, M. D. [Published in the British and Foreign Medico-Chirurgical Review, for January, 1853.]

Dr. Budd, in his preface to M. Melsens' Memoir, very justly observes, that, "if as M. Melsens alleges, and as his numerous experiments seem conclusively to show, iodide of potassium is not only a safe, certain and radical cure for the common forms of saturnine and mercurial poisoning, but an equally sure preventive of the injurious effects so frequently produced by emanations from lead and mercury, the fact is one that cannot be made too widely known." And M. Melsens has attempted further to establish a relation between the iodide of potassium and mercury, as therapeutic agents, which is no less deserving of attention.

His starting point in reference to mercurial and saturnine poisoning is, that the metallic substance is in actual union with the affected part or parts, and is retained there in the form of some *insoluble* compound. "The iodide of potassium, after its absorption into the blood, combines with the metallic poison, and forms with it a new and *soluble* salt—liberates the poison from its union with the injured part—dissolves it out, so to speak, from the damaged fibre, and sets it once more afloat in the circulation." And the double iodide of mercury (or lead) and potassium is assumed to be eliminated through the kidneys almost as soon as formed, with any excess of iodide of potassium present.

The modus operandi of the antidote proposed here is apparently in antagonism with established views of antidotical action. In attacking a poison in the stomach, our purpose is to convert it from a soluble to an insoluble compound, with the aim of preventing its absorption into the circulation. But the object here is the reverse, to restore the poison to the current of the circulation, and cause its final elimination by uniting it with a substance readily cast out of the system.

It appears, from the experiments of M. Melsens, that the *kidneys* are the principal outlet for the iodide of potassium from the system ; that, under ordinary circumstances, it is seldom or never traced in the fecal evacuations ; and that even when associated with active purgatives, it is with extreme difficulty made to pass through the bowels into the stools. So much for the channel of elimination. The essential chemical point in the eliminative process is the fact that all the mercurial and saturnine compounds which can possibly occur in the economy, are soluble in the iodide of potassium; the presence of the organic substances of the body not hindering these reactions. We quote at length M. Melsens' views and statements on these questions; and first as to mercury:

"It is well known that persons who have undergone mercurial treatment have observed, even after an interval of many years, that gold placed in contact with their perspiration has become coated with mercury, especially when excessive perspirations have been caused by the use of the vapor bath. If this fact be true, it proves that the system may absorb and retain mercury for a long time under forms which I will not try to define, but which very probably result from the insoluble compounds which the salts of mercury form, either with the organic or inorganic materials of the body, or with both conjoined. Perhaps the mercury may even occur in the metallic state, as some have admitted; at any rate, it is present in the body in such form as to be retained there. The principal combinations which might thus occur may be reduced to the following:

1. Combinations of corrosive sublimate, whether in its simple state, or as modified by the animal substances of the economy—namely,
   *a.* With albumen.
   *b.* Albumen, and the materials of the brain.
   *c.* Gelatine.
   *d.* The nitrogenous extractive matters of the blood, of muscle, of the urine, &c.
   *e.* Albumen, fibrin, muscular fibre, pelatine, whether in the natural state or modified by digestion.
   *f.* Matters of the bile.
2. Mercurial soaps.
3. Phosphates of mercury.
4. Mercury in the metallic state. (?)

All these compounds are soluble in alkaline or neutral pure iodide ot potassium dissolved in one of the liquids of the body. I have made experiments with each of the compounds here enumerated, and have always succeeded in dissolving them under whatever circumstances. If the iodide of potassium be associated with a dilute acid which has no energetic action either on the solution of the salt or on the principles which occur in the body, the solution of the fixed mercurial compounds is still effected perfectly. In obtaining this last result I operated with lactic acid. After fixing corrosive sublimate on nervous filaments, muscular fibre, or on tendons, it is only necessary to wash them for a short time in a solution of iodide of potassium, whether acid, alkaline, or neutral, in order to remove entirely the mercurial salt. It is especially with a solution of albumen and sublimate that these properties admit of being perfectly demonstrated; indeed, the experiment has been for many years a class experiment in M. Dumas' course at the School of Medicine. It is only necessary to pour a solution of iodide of potassium on the precipitate formed by albumen and sublimate, in order to see the liquid become instantly limpid. The iodide of mercury possesses a property which I must not omit to point out. This compound, as is well known, is soluble in caustic potash. Now, although caustic potash is not found in the living body, the alkalinity of the greater number of the greater of the fluids which are found there is worthy of being borne in mind, and acquires a certain degree of interest when viewed in relation to the following experiment. Mercury coarsely divided was placed under a layer of water holding in solution caustic potash and iodide of potassium. After some weeks' contact a considerable quantity of mercury was found dissolved in the liquid.

It is well known that metallic mercury in contact with alkaline chlorides in solution, itself passes in part to the state of chloride. It remained for me to prove that this reaction might occur in perfectly neutral or even in alkaline fluids. It was necessary in this experiment to guard against the intervention of the carbonic acid of the air, or of the acids sometimes diffused in the air of chemical laboratories. It appeared to me quite necessary to make this experiment, especially as it was possible that mercury might exist in the body in the metallic state, and yet act as a poison; in which case, the experiment I have just cited would still permit the hope that the poison might become dissolved by the action of the iodide of potassium, rendered alkaline by the fluids of the living body.

When metallic mercury is shaken in a solution of iodide of potassium, whether neutral or slightly acidulated with hydrochloric acid, the solution soon acquires an alkaline reaction—an incontestable proof that the oxygen of the air has laid hold of the potassium of the iodide, which, in its turn, has yielded its iodine to the mercury, the iodide of mercury thus formed having entered into combination with the iodide of potassium remaining in excess. It suffices to shake vigorously a neutral or slightly acidulated solution of iodide of potassium with an excess of mercury, for the space of a minute, in order to see the reaction occur. This might be easily done as a class-experiment, to show the tendency of the alkaline haloid salts to form double salts with the corresponding haloid salts of the metals properly so called. An analogous phenomenon may be observed, although less readily, with common salt, and, indeed, simple potash excites the oxidation of mercury, and dissolves small quantities of the oxide. When these experiments are made in closed vessels, it is easy to demonstrate the disappearance of oxygen, by analysing the air which has been operated on."

The solubility of the compounds of lead in iodide of potassium, is, it is admitted, of more difficult proof. On this point he says:

"I shall content myself with observing, therefore, that the iodide of lead is soluble in alkaline liquids, and that it has a marked tendency to combine with alkaline iodides. These facts have appeared to me to constitute adequate motives to induce medical men to employ the treatment by iodide of potassium for complaints which, however much they may be relieved, are, according to our best physicians, rarely, if ever, radically cured.

"I have proved that metallic lead becomes dissolved in a solution of iodide of potassium, rendered alkaline by potash. It is well known how rapidly certain metals become oxidized when exposed to moist air, in the presence of even feeble acids, like the carbonic acid of the atmosphere. This is the case with iron, and indeed with lead. The intervention of an alkali sometimes suffices to prevent oxidation. Such is the case with iron. Lead, copper, and zinc become oxidized, on the contrary, more rapidly in contact with an alkaline liquor. I have proved that granules of perfectly metallic lead, bathed by a solution of iodide of potassium, rendered alkaline by potash, become, after a time, partly dissolved in this mixture."

The legitimate sequitur of these chemical reactions is obviously increased activity of certain salts of mercury in the stomach, when followed by the administration of iodide of potassium. And this point seems to be made out both by reference to the statements and experiments of other chemists, and to M. Melsens' own researches with particular reference to the subject. And as a confirmatory fact, it is asserted that, in the treatment of secondary syphilis, "the administration of iodide of potassium often causes intense suffering in patients who have been treated by mercurials."

Analogous results are shown to have occurred after the administration of iodide of potassium to animals who have taken lead compounds—that is, aggravation of the poisonous phenomena. And, hence, it is urged that "great caution is necessary in the employment of this remedy in man, for the first few days." For, "at the moment when the metallic compounds fixed in the body become dissolved or transformed, phenomena of acute poisoning may occur, caused by their liberation."

Numerous cases are adduced of the successful application of this treatment to the relief of lead poisoning and mercurial poisoning. In fact, it is impossible to disbelieve in the remedial value of the agent, after perusing the mass of testimony by which it is attested. We confess, however, that plausible as is the theory of its action, we do not clearly understand the rapidly succeeding activity and disappearance from the system which we are asked to admit in the combination of the poison and antidote.

---

*A Discourse on the Times, Character and Writings of Hippocrates, read before the Trustees, Faculty, and Medical Class of the College of Physicians and Surgeons, at the opening of the term*, 1852–53. By ELISHA BARTLETT, M. D., Professor of Materia Medica, &c. New York, 1852.

This elegant and instructive picture of the life and times of the Father of Medicine, will be read with general interest, and must take a place in our permanent medical literature.

---

*The Physician's Pocket Dose and Symptom Book; containing the Doses and Uses of all the principal Articles of the Materia Medica and Chief Officinal Preparations, &c. &c.* By JOSEPH H. WYTHES, M. D. Philadelphia, Lindsay & Blakiston: 1853. pp. 246.

This will be found an exceedingly convenient little pocket manual, especially useful to the young practitioner, in the emergencies which he so often encounters in the commencement of practice.

---

*What to observe at the Bed-Side and after Death in Medical Cases.* Published under the authority of the London Medical Society of Observation. Philadelphia: Blanchard and Lea. 1853.

This little book strikes us as meeting a want that has long been felt—in offering suggestions for uniformity and precision in the observation of disease. The form of arrangement of symptoms and after-death appearances, appears to have been originally framed by Dr. Walshe, and thrown into its present shape by a Committee of the London Medical Society of Observation, under the supervision of Dr. Ballard. These names are a guarantee for the thorough execution of the plan; and a rapid glance at the work has impressed us most favorably of the completeness with which it has been accomplished.

*Introductory Lecture to the Course of Chemistry at the Jefferson Medical College.* By Franklin Bache, M. D.

*Introductory Lecture to the Course of Chemistry at the Pennsylvania Medical College.* By John J. Reese, M. D.

We have to acknowledge with pleasure the receipt of these very able and instructive introductories to the branch of Chemistry, in the institutions designated. Dr. Bache's eloquent discourse cannot fail to sustain his high reputation as a Chemist and Teacher: and Dr. Reese's maiden effort is an appropriate introduction to the successful course which, we learn, he has delivered.

